# Human level information extraction from clinical reports with finetuned language models

**DOI:** 10.1038/s41598-025-28767-z

**Published:** 2025-11-24

**Authors:** Longchao Liu, Long Lian, Yiyan Hao, Aidan Pace, Elaine Kim, Nour Homsi, Yash Pershad, Liheng Lai, Thomas Gracie, Ashwin Kishtagari, Peter R. Carroll, Alexander G. Bick, Anobel Y. Odisho, Maggie Chung, Adam Yala

**Affiliations:** 1 Electrical Engineering and Computer Sciences, UC Berkeley, 387 Soda Hall, Berkeley, CA 94720 USA; 2 Department of Bioengineering, University of Pennsylvania, 210 South 33rd St, Philadelphia, PA 19104 USA; 3 University of California San Francisco School of Medicine, 533 Parnassus Ave, 94143 San Francisco, CA USA; 4 University of California Riverside School of Medicine, 92521 Botanic Gardens Dr, Riverside, CA 92507 USA; 5 Division of Genetic Medicine, Department of Medicine, Vanderbilt University Medical Center, 2213 Garland Avenue, Nashville, TN 37232 USA; 6 Department of Medicine, Vanderbilt University Medical Center, 2213 Garland Avenue, Nashville, TN 37232 USA; 7 Vanderbilt Ingram Cancer Center, Vanderbilt University Medical Center, 2220 Pierce Ave, Nashville, TN 37232 USA; 8 Department of Urology, School of Medicine, University of California San Francisco (UCSF), 1825 4th St, Box 1695, 94143 San Francisco, CA USA; 9 Computational Precision Health, UC Berkeley and UCSF, 2177 Hearst Ave, Berkeley, CA 94709 USA; 10 Department of Radiology and Biomedical Imaging, School of Medicine, University of California San Francisco (UCSF), 505 Parnassus Ave, 94143 San Francisco, CA USA; 11 Department of Statistics, UC Berkeley, 367 Evans Hall, Berkeley, CA 94720 USA

**Keywords:** Deep learning, Pathology, Informatics, Computational models, Data processing, Machine learning

## Abstract

Extracting structured data from clinical notes remains a key bottleneck in clinical research. We hypothesized that with minimal computational and annotation resources, open-source large language models (LLMs) could create high-quality research databases. We developed Strata, a low-code library for leveraging LLMs for data extraction from clinical reports. Trained researchers labeled four datasets from prostate MRI, breast pathology, kidney pathology, and bone marrow (MDS) pathology reports. Using Strata, we evaluated open-source LLMs, including instruction-tuned, medicine-specific, reasoning-based, and LoRA-finetuned LLMs. We compared these models to zero-shot GPT-4 and a second human annotator. Our primary evaluation metric was exact match accuracy, which assesses if all variables for a report were extracted correctly. LoRa-finetuned Llama-3.1 8B achieved non-inferior performance to the second human annotator across all four datasets, with an average exact match accuracy of 90.0 ± 1.7. Fine-tuned Llama-3.1 outperformed all other open-source models, including DeepSeekR1-Distill-Llama and Llama-3-8B-UltraMedical, which obtained average exact match accuracies of 56.8 ± 29.0 and 39.1 ± 24.4 respectively. GPT-4 was non-inferior to the second human annotator in all datasets except kidney pathology. Small, open-source LLMs offer an accessible solution for the curation of local research databases; they obtain human-level accuracy while only leveraging desktop-grade hardware and ≤ 100 training reports. Unlike commercial LLMs, these tools can be locally hosted and version-controlled. *Strata* enables automated human-level performance in extracting structured data from clinical notes using ≤ 100 training reports and a single desktop-grade GPU.

## Introduction

Artificial intelligence (AI) tools in medicine aim to improve patient care by learning patterns across massive, well-annotated datasets. These annotations, describing key patient characteristics, are often derived from clinical notes, such as pathology and radiology reports. Extracting ground-truth labels from clinical notes remains a critical research bottleneck. As a result, our study focuses on research database curation, namely the systematic process of turning massive collections of clinical notes into clean tabular datasets. The traditional approach to database curation is manual chart review, where researchers annotate thousands of reports. If the research team wishes to add a new variable to their database, a new round of exhaustive annotation is required. It is intractable to curate health-system scale research databases with retrospective manual annotation. There is an unmet need for scalable and human-level data extraction from clinical reports with minimal engineering overhead.

Automated tools, including deep learning^1,2^, statistical machine learning^3–6^, and rule-based^7–9^ methods, have been proposed to extract structured information from clinical reports. While these systems have achieved promising performance, they require substantial effort to develop and deploy. As a result, their adoption by the research community remains limited. Large language models (LLMs) have demonstrated exciting capabilities in general text understanding, detecting errors in radiology reports^10^, streamlining radiology report impressions^11,12^, and automatic synoptic reports^13^. Recent works^14,15^ present powerful methods for data-scarce few-shot learning, particularly in relation extraction and named entity recognition tasks. However, they require non-trivial model customization and pretraining strategies. Ideally, researchers could structure information extraction as a question-answering task using a shared, minimal-engineering setup. Commercial LLMs offer promising capabilities, yet they are ill-suited for health-system-scale research database curation due to reproducibility, cost, and privacy concerns. Commercial LLMs are continually updated, and if new versions of the LLM perform unexpectedly worse given the same inputs, it can be infeasible to reproduce prior extractions after model updates. Furthermore, costs associated with using commercial LLMs can pose significant barriers, and institutional agreements are often required to address privacy concerns. Ideally, researchers could locally host their LLMs (“self-hosting”) on cheap “desktop-grade” hardware; these local LLMs would enable reproducible research while minimizing costs and avoiding the transfer of sensitive data.

Open-source LLMs, such as Llama^16–18^ and Mistral^19^, offer a promising solution for efficient “self-hosted” information extraction. Small LLM variants (i.e., < 11 billion parameters) with instruction-tuning allow for efficient general text understanding and cheap customization. Recent medically fine-tuned LLMs, including PMC-Llama 13B and Lama-3-8B-UltraMedical, have demonstrated compelling performance on biomedical question-answering benchmarks^20,21^. Small versions of the DeepSeek-R1 model have demonstrated strong general reasoning performance while building on small-scale general LLM backbones^22^, Llama-8B and Qwen-2.5-7B. While these LLMs offer exciting performance across general benchmarks, it remains unclear how these tools perform in clinical information extraction.

**Fig. 1:**
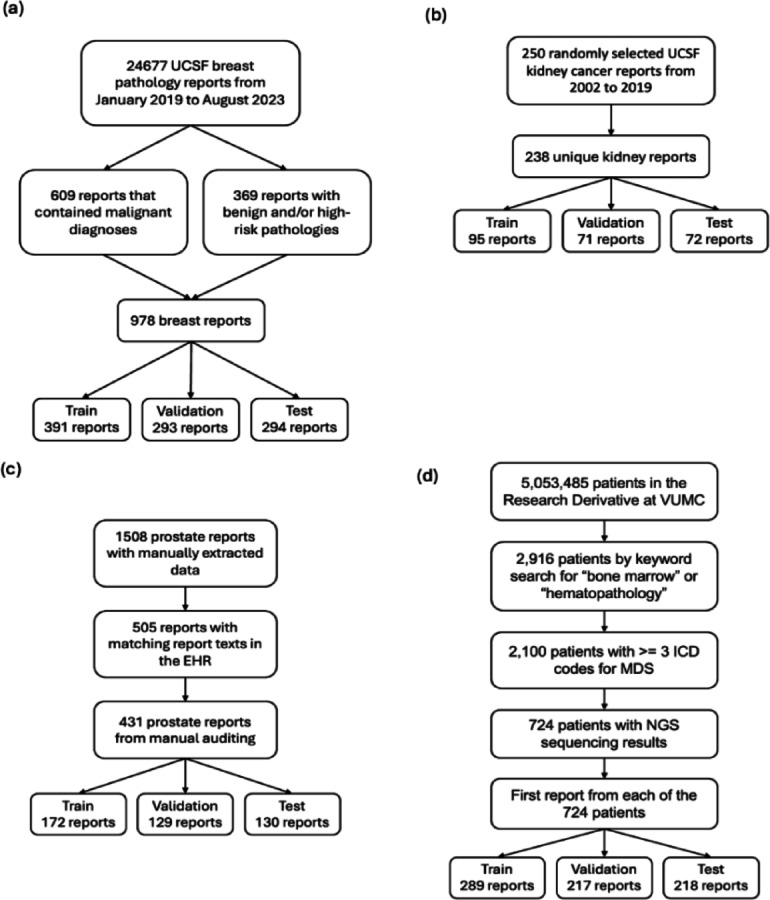
Dataset construction flowchart for our breast (**a**), kidney (**b**), prostate (**c**), and MDS (**d**) datasets. UCSF = University of California, San Francisco, VUMC = Vanderbilt University Medical Center, EHR = electronic health records, MDS = Myelodysplastic Syndrome.

In this study, we focus on empowering individual researchers to curate large-scale research databases at their institution, using minimal computing (i.e., a few hours on a single GPU) and annotation resources (i.e., < 100 reports annotated). Our strategy aligns with the practical constraints and foundational research workflows of academic medical centers. Following this strategy, we do not develop massive 400B + open-source LLMs that require multiple H100 servers to host or generic universal LLMs that can generalize across institutions. We hypothesized that with simple fine-tuning techniques, small-scale open-source LLMs could enable individual researchers to create high-quality datasets customized to their institution, empowering their downstream AI research. Our extraction quality goal is “human-level performance”; specifically, we test the ability of LLMs to achieve statistically non-inferior performance to a medical student extracting the same information from clinical notes. The comparison to medical students is not meant to suggest equivalence to specialist performance, but rather to assess whether LLMs can match the standard level of accuracy currently used to curate large-scale research databases. We benchmark a wide range of “self-hosted” LLM approaches on four diverse real-world datasets from ongoing AI research projects at our institutions. To facilitate the broader adoption of customized open-source LLMs, we release a low-code library, Strata, that supports the fine-tuning, evaluation, and deployment of LLMs for clinical information extraction (Fig. 1).

## Materials and methods

We collected four diverse clinical text datasets underlying active research projects at our institutions across breast, kidney, prostate, and bone marrow cancer (Myelodysplastic Syndrome, MDS). These real-world datasets represent our intended use cases, where LLM-based tools empower the automatic creation of large-scale structured datasets to support downstream research at a single medical center. Our breast, kidney, and prostate datasets were annotated by medical students, and our MDS dataset was annotated by a hematologist. The medical students were specifically trained for the task by attending physicians with 8 to 11 years of post-graduate clinical experience. We describe each dataset in detail, including its creation and annotation process, in Appendix A1 of our supplementary methods.

### Extracting structured information using customized large Language models

**Fig. 2:**
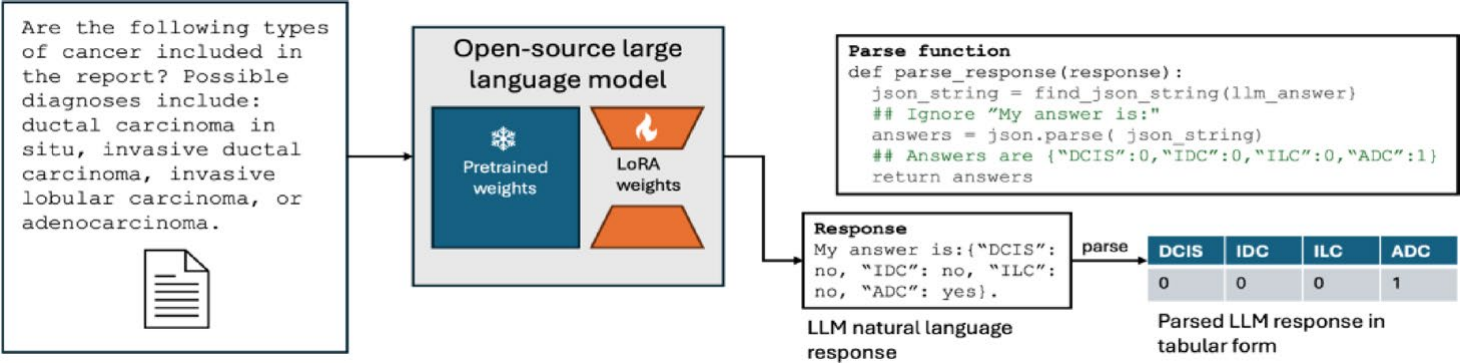
Process for leveraging open-source LLMs to extract structured information from clinical reports. The input to the LLM consists of a user prompt, which specifies the task (e.g., cancer subtyping), paired with the report text. An open-source LLM contains pre-trained weights (e.g., 8B parameters for Lamma-3.1 8B) learned across web-scale datasets; when fine-tuning an LLM, we introduce additional LoRA weights (~ 400 M parameters) to customize the model. The model generates an answer in text, parsed by a user-defined “parse function” into a tabular form.

State-of-the-art LLMs are trained to follow instructions specified in text prompts when generating their answers. This process, called instruction-tuning^23^, enables users to define tasks with instruction prompts. To leverage instruction-tuned LLMs for clinical information extraction, a user can write out the desired task (e.g., extract the cancer subtype from a pathology report) in a text prompt before inputting the full clinical report. Given the prompt and the clinical report, the LLM will generate an answer in the text format, which can be parsed into the desired structured format using a simple post-processing script. This process is illustrated in Fig. 2.

**Fig. 3:**
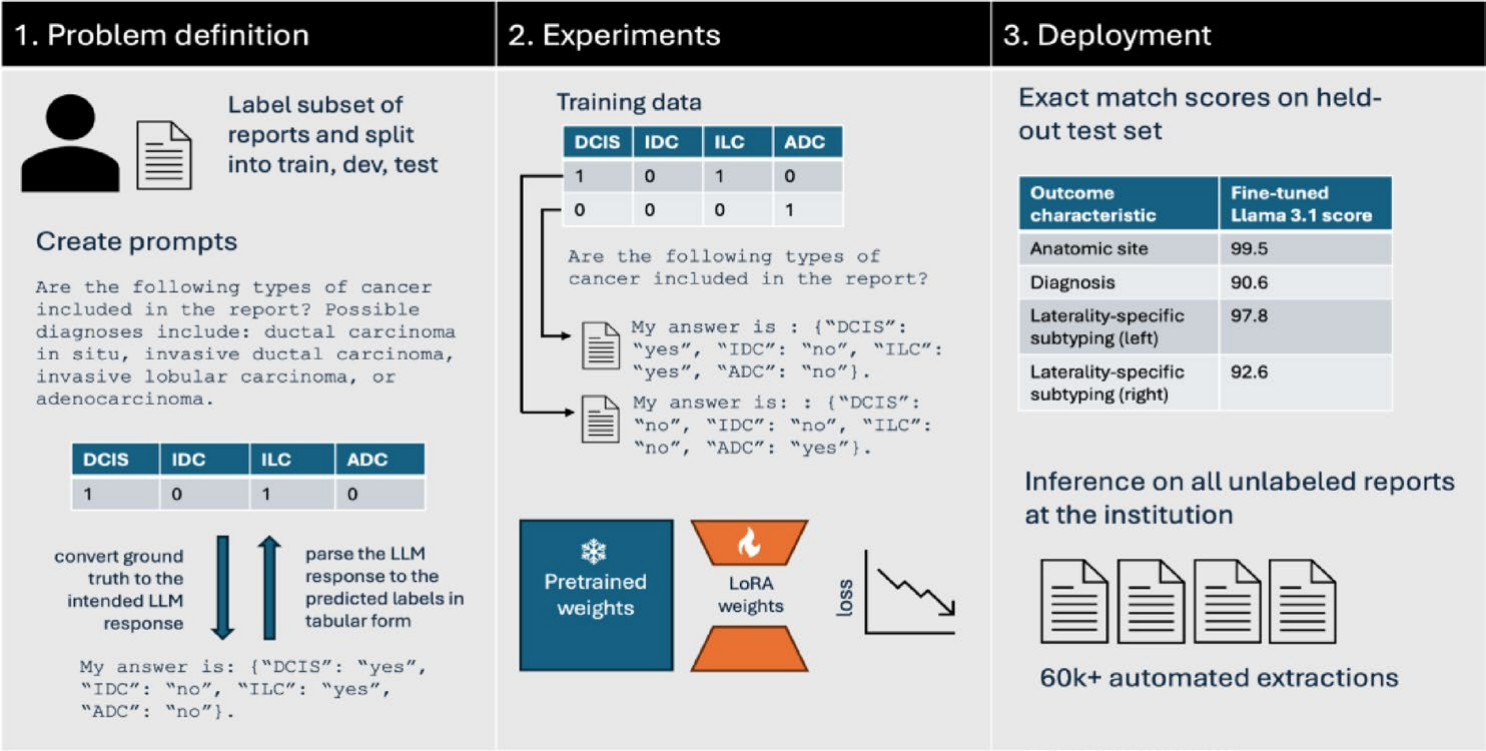
Process of using Strata to create research databases. The researcher labels a subset of the reports, defines prompts, and creates parsing functions to interpret the model’s response. Given this information, Strata supports LLM fine-tuning and deployment. In LoRA fine-tuning, the model’s LoRA weights are trained to generate the templated answers. Strata supports model evaluation and efficient bulk-inference. Bulk-inference is used to create a research database from all unlabeled reports at the institution.

In this work, we are interested in customizing local LLMs for clinical information extraction. To enable the full research community to benefit from this resource, we focus on LLMs that can be easily hosted on a single consumer desktop-grade GPU, such as the Nvidia RTX 3090 or 4090. As a result, we leverage three classes of small LLMs: (1) medically fine-tuned QA models, including PMC-LLaMA 13B and Llama-3-8B-UltraMedical, (2) accessible versions of leading reasoning models, including DeepSeek R1 distilled to Llama-3.1 8B and Qwen2.5-Math 7B, and (3) popular base instruction-tuned models, such as Llama-3.1 8B, Gemma-2-9B, and Mistral-v0.3 7B. These billion-parameter LLMs are orders of magnitude smaller than state-of-the-art LLMs such as Llama-3.1 405B and GPT-4. To enable smaller LLMs to achieve human-level performance in clinical information extraction, we fine-tune them. Specifically, we leverage simple preprocessing scripts to convert human-annotated structured labels (i.e., in a tabular format) to text answers for the LLM to generate (e.g., “My answer is: {“DCIS”: “yes”, “ILC”: “no”}”). Given a dataset of pairs of clinical reports and answer texts, we can train the LLM to correctly predict the intended answer from the user prompt and clinical report. This process is illustrated in Fig. 3. To fine-tune LLMs using desktop-grade hardware, we employ Low-Rank Adaption (LoRA)^24^. Instead of directly optimizing the billions of parameters in our LLMs, LoRA keeps the initial weights of the LLM frozen and adds a small number of new parameters (e.g., < 400 M parameters) across the LLM to customize the model’s behavior.

To enable the broader clinical and research communities to fine-tune and deploy their LLMs on local hardware, we developed *Strata*, a lightweight library for clinical information extraction. As illustrated in Fig. 3, Strata is used in three phases. First, researchers annotate a small dataset of clinical reports with their desired structured labels, which are partitioned into a training, development, and testing set. In addition to these annotations, researchers define the task for the LLM by specifying a prompt, a parsing script, and a template function. As described above, the parsing script maps the LLM’s answer to the desired structured format, and the template function maps the structured annotation to a text-based answer. Given an annotated dataset, Strata supports LoRA-based fine-tuning and flexible hyperparameter optimization. Finally, Strata offers a rich automated evaluation suite for model selection. We leverage Strata across all our experiments.

### LLM development and evaluation

Across all datasets, we developed prompts, parsing scripts, and template functions to maximize the performance of our open-source LLMs on the development sets. In these experiments, the models were used without fine-tuning (i.e. zero-shot). For the DeepSeek-R1 models, we modified the prompts to allow for additional reasoning preceding the final answer, as shown in Supplementary Table S1. For evaluation, we ensure that responses are complete in Supplementary Table S2, then explicitly excluded the reasoning content between the “Think:” and “Answer:” tags and used only the final output in our accuracy calculations. This ensured that DeepSeek was evaluated on the same basis as other models and avoided penalizing models for verbosity or non-standard formatting in intermediate steps. Given a final set of prompts and scripts, we used LoRA to fine-tune our best-performing non-reasoning model, Llama-3.1. We show detailed examples of templates and responses in Appendix A2.

LLM fine-tuning requires selecting multiple hyperparameters, which leads to a variety of design choices on model architecture, data augmentation, and training recipes. LoRA includes two hyperparameters, namely rank and alpha. LoRA rank determines the size of the trainable parameters in fine-tuning, and alpha scales the output of these trainable layers relative to the original model’s outputs. In the context of the MDS dataset, we also experimented with class balancing, where rare values are oversampled during training. Finally, we also tuned the number of training epochs and the learning rate of the optimizer. Using Strata, we performed a grid search over possible hyperparameters in mixed-dataset fine-tuning on the development set. We applied the optimal hyperparameters in all other settings.

We considered three possible variants of LLM fine-tuning. First, we trained a customized LLM for each task within each dataset separately (i.e., question-specific fine-tuning). Next, we explored dataset-specific fine-tuning, in which all tasks within a single dataset (e.g., specimen site, cancer presence, and subtyping) were included in fine-tuning a model. Finally, we also considered training a single fine-tuned LLM across all datasets and tasks (i.e., mixed-dataset fine-tuning).

We leverage the annotations of our first human annotator as our reference labels in all evaluations. Our primary evaluation metric is “exact-match accuracy,” where we evaluate the ability of the LLM to extract all components of user-specified queries correctly. For instance, if the reference (i.e., first human annotator) response for breast cancer subtyping were “{DCIS: Yes, ILC: No, IDC: Yes},” the LLM predictions would have to match across all fields to gain a point in our cancer subtyping exact-match accuracy calculation. For dataset-level exact match accuracies, the LLM must correctly extract the information across all user queries for that clinical report. In addition to our stringent exact match accuracies, we also evaluate average accuracies and macro-F1, precision, and recall scores in our Supplementary Tables S3, S4, and S5.

We compare our open-source LLM results to GPT-4 and a second human annotator. All LLMs and the second human annotator were given the same prompts, and their extractions were compared to the first human annotator extractions as reference labels, using the metrics described above. The accuracy of our second human annotator serves as our benchmark for “human-level performance”. We hypothesize that fine-tuned LLMs would be non-inferior to the second human annotator in clinical information extraction. Our goal is not to outperform the second human annotator in matching the first human annotator; we expect disagreements between human annotators to reflect ambiguity in clinical reports. The second human annotator for each test set was a medical student who received training on the relevant pathology classifications and the specific criteria for annotation for each dataset. Both annotators receive appropriate training, so each of their performance individually is on par with what we would expect in real-world database curation. We compare the labels of the two readers using Cohen’s kappa coefficient and inter-annotator agreement rates.

To understand the dependence of fine-tuned LLM performance on labeled data, we performed learning curve analyses. We randomly sampled subsets of each training set (consisting of 25% and 50% of the full training data) and fine-tuned using only the subsets at the dataset-specific level. We then evaluated the model performance with different subsets against the second human annotator to determine the number of labeled reports needed for human-level performance.

To understand the effect of hyper-parameters on our performance, we performed a sensitivity analysis. For each hyperparameter (i.e., LoRA rank, LoRA scaling, number of epochs, and learning rate), we varied one hyperparameter value while holding the others constant. For all fine-tuning experiments, we used a linear learning rate scheduler with a warmup of 5 steps. The learning rate increases during the warmup phase and then decays linearly over the remainder of training. We did not perform additional tuning of the learning rate schedule. This supports our broader aim of evaluating how well models perform under minimal configuration. In particular, this setup offers a robust and practical starting point for low-code workflows, where researchers can fine-tune models with minimal effort and domain-specific engineering. We evaluated the difference in exact match accuracy.

### Statistical analysis

We used scikit-learn (version 1.5.1; *scikit-learn.org*) and SciPy (version 1.14.1; *scipy.org*) to compute all evaluation metrics, including F1-score, precision, and recall. We computed evaluation metrics across 5000 bootstrap samples (16) to obtain 95% confidence intervals (CIs). To compare the exact match accuracy of LLMs to the accuracy of a second human annotator, we utilized a non-inferiority t-test using the stats R package (version 4.4.1; *cran.r-project.org*). Given the size of each test set, 80% desired power, a 0.05 p-value, and the standard deviation of the second human annotator accuracy, we computed the margin of error measurable by each test set. This power calculation was performed using the pwr R package (version 1.3-0; *cran.r-project.org*), and we utilized dataset-specific margins in each non-inferiority calculation. Given 294, 130, 72, and 218 reports in the breast, prostate, kidney, and MDS test sets, this resulted in non-inferiority margins of 6.3%, 8.2%, 13.5%, and 5.9%, respectively.

### Ethical compliance

This study was approved by the University of California, San Francisco (UCSF) Institutional Review Board. The requirement for written informed consent was waived by the UCSF Institutional Review Board. All experiments were conducted in accordance with the relevant guidelines and regulations.

## Results

### Report characteristics

Our breast, kidney, prostate, and MDS datasets contained 978, 238, 431, and 724 reports (Fig. 1). Of these reports, 294, 72, 130, and 218 were randomly selected for held-out testing for breast, kidney, prostate, and MDS extractions, and these reports contained an average of 766, 688, 423, and 785 words, respectively. Amongst breast, kidney, and prostate test sets, 65%, 94%, and 85% respectively contained a confirmation of cancer. 7% of the MDS test set confirmed untreated MDS. We report detailed text and label statistics for all four datasets in Table 1.

**Table 1:** Detailed outcome and text characteristics for each Dataset.

Outcome Characteristic	Train	Val	Test
Breast	*n* = 391	*n* = 293	*n* = 294
No. of words mean ± SD (min-max)	809 ± 677 (200–3596)	733 ± 579 (189–3991)	766 ± 640 (183–3717)
Anatomic Site			
Right breast	222 (57%)	162 (55%)	166 (56%)
Left breast	213 (54%)	158 (54%)	150 (51%)
Right lymph node	41 (10%)	30 (10%)	336 (114%)
Left lymph node	41 (10%)	29 (10%)	34 (12%)
Diagnosis			
Cancer	242 (62%)	177 (60%)	190 (65%)
High risk	31 (8%)	29 (10%)	23 (8%)
Benign	118 (30%)	87 (30%)	81 (28%)
Laterality-specific subtyping			
Ductal carcinoma in situ	142 (36%)	109 (37%)	121 (41%)
Invasive ductal carcinoma	109 (28%)	91 (31%)	109 (37%)
Invasive lobular carcinoma	25 (6%)	16 (5%)	12 (4%)
Adenocarcinoma NOS	12 (3%)	12 (4%)	5 (2%)
Kidney	*n* = 95	*n* = 71	*n* = 72
No. of words mean ± SD (min-max)	768 ± 412 (270–3081)	741 ± 361 (275–2081)	688 ± 259 (128–1652)
Cancer Diagnosis			
Yes	84 (88%)	62 (87%)	68 (94%)
No	11 (12%)	9 (13%)	4 (6%)
Cancer Anatomic Site			
Left lower pole	19 (20%)	12 (17%)	11 (15%)
Right lower pole	10 (11%)	8 (11%)	11 (15%)
Left middle pole	11 (12%)	14 (20%)	11 (15%)
Right middle pole	17 (18%)	10 (14%)	15 (21%)
Left upper pole	17 (18%)	17 (24%)	16 (22%)
Right upper pole	13 (14%)	10 (14%)	13 (18%)
Prostate	*n* = 172	*n* = 129	*n* = 130
No. of words mean ± SD (min-max)	431 ± 131 (110–916)	436 ± 176 (166–1587)	423 ± 109 (172–699)
PIRADS			
5	58 (34%)	32 (25%)	43 (33%)
4	69 (40%)	54 (42%)	46 (35%)
3	15 (9%)	18 (14%)	20 (15%)
2	14 (8%)	12 (9%)	12 (9%)
1	3 (2%)	2 (2%)	0 (0%)
Cancer Diagnosis			
Yes	149 (87%)	108 (84%)	111 (85%)
No	23 (13%)	21 (16%)	19 (15%)
MDS	*n* = 289	*n* = 217	*n* = 218
No. of words mean ± SD (min-max)	810 ± 222 (219–1652)	805 ± 234 (171–1725)	785 ± 225 (151–1612)
Bone marrow			
Yes	254 (88%)	195 (90%)	193 (89%)
No	35 (12%)	22 (10%)	25 (11%)
Untreated Myelodysplastic Syndrome			
Yes	19 (7%)	18 (8%)	16 (7%)
No	235 (81%)	177 (82%)	177 (81%)
Missing	35 (12%)	22 (10%)	25 (12%)

Unless otherwise specified, data are numbers of reports, with percentages for each split in parentheses. For breast and prostate, “laterality-specific” means that the model was asked separately for each laterality (see Table 2). For diagnosis characteristics, the outcomes sum to 100%. For anatomic site and subtyping, the outcomes are not mutually exclusive, so the outcomes sum to > 100%. NOS = not otherwise specified.

### Inter-annotator analysis

We report our prompts for each dataset and task in Table 2 and a detailed model performance evaluation in Table 3. Our primary performance metric, exact match accuracy, evaluates the ability of a model to correctly extract all fields for a report, using the labels of the first human annotator as the reference. Our second human annotators obtained exact match accuracies of 75.5 (95% CI 70.1, 80.3), 83.3 (95% CI 73.6, 90.3), 83.1 (95% CI 76.2, 88.5), 85.8 (95% CI 80.7, 89.9) in the breast, kidney, prostate, and MDS test set, respectively. These correspond to average Cohen’s kappa coefficient scores of 0.87, 0.78, 0.88, and 0.54 for each dataset and inter-annotator agreement among 97.3%, 94.0%, 93.3%, and 92.4% of fields. This extraction-level error rate aligns with prior studies on medical record abstraction, which report error rates ranging from 0.7% to 20.8%^25^. The inter-annotator agreement sources of disagreement are reported in Supplementary Figure S1.

**Table 2:** Task-specific LLM prompts for each Dataset.

Breast	
Anatomic Site	What are the tissue sources that are investigated in this report? Your answer can include “left breast”, “right breast”, “left lymph node”, or “right lymph node”. If multiple sources are present, list all of them and separate them with a comma. Do not provide explanations. Do not output anything other than the four choices.
Diagnosis	Is there cancer diagnosed in this report? If so, what is the laterality of the cancer? Your answer should be in json format: {“left”: __, “right”: __}, where each blank is filled in by “1” if any cancer is deterministically diagnosed (i.e. DCIS, IDC, ILC, other invasive tumors, adenocarcinoma), “0” if the tissue is benign, or “2” if there are no cancers but high risk lesions identified explicitly. High risk lesions are defined as any of the following: atypia, radial scar, complex sclerosing lesion, atypical ductal hyperplasia, lobular carcinoma in situ, atypical lobular hyperplasia, FEA, ALH, ADH, phyllodes. If no breast or axillary lymph node is examined for a laterality, or if the report is about other organs (e.g. ovaries, liver, bone, adnexa, omentum, or lymph nodes in the abdomen/pelvis), fill in the corresponding blanks with “N/A”. Provide your answer in the required format. Do not provide explanations or output anything else.
Laterality specific subtyping (left)	Identify which laterality of tissues are examined in this report. If the left breast is not examined in this report, output “N/A” and stop generating immediately, ignore the following prompts. Proceed to answer the following question only if the left breast is examined: Based only on the part of the report that is about the left breast, are the following type(s) of cancer diagnosed in the left breast? Possible diagnoses include: “DCIS” for ductal carcinoma in situ, “IDC” for invasive ductal carcinoma, “ILC” for invasive lobular carcinoma, or “adenocarcinoma” for adenocarcinoma. Provide your answer in json format: {“DCIS”: __, “IDC”: __, “ILC”: __, “adenocarcinoma”: __} Fill in the corresponding blank with 0 if no such cancer is diagnosed, 1 if such cancer is diagnosed in the left breast. Provide your answer in the given json format. Do not provide explanations. Do not include information about the right breast.
Laterality specific subtyping (right)	Identify which laterality of tissues are examined in this report. If the right breast is not examined in this report, output “N/A” and stop generating immediately, ignore the following prompts. Proceed to answer the following question only if the right breast is examined: Based only on the part of the report that is about the right breast, are the following type(s) of cancer diagnosed in the right breast? Possible diagnoses include: “DCIS” for ductal carcinoma in situ, “IDC” for invasive ductal carcinoma, “ILC” for invasive lobular carcinoma, or “adenocarcinoma” for adenocarcinoma. Provide your answer in json format: {“DCIS”: __, “IDC”: __, “ILC”: __, “adenocarcinoma”: __} Fill in the corresponding blank with 0 if no such cancer is diagnosed, 1 if such cancer is diagnosed in the right breast. Provide your answer in the given json format. Do not provide explanations. Do not include information about the left breast.
Kidney	
Cancer Diagnosis	Based on information on the report, is there cancer diagnosed in the kidney? If this is not a kidney pathology report, answer ‘no’. If the cancer is diagnosed in other organs outside of the kidney, answer ‘no’. Possible cancer types include but is not limited to: renal cell carcinoma, collecting duct carcinoma, oncocytoma, and renal medullary carcinoma. Keep in mind that angiomyolipoma is not a cancer. Choose your answer from ‘yes’ or ‘no’. Provide your answer as one word only, do not provide explanations.
Cancer Anatomic Site	Where in the kidney is the cancer located based on this report, if any? If there is no cancer diagnosed in the kidney, answer ‘N/A’ and stop generating. Choose your answer from ‘left lower pole’, ‘right lower pole’, ‘left middle pole’, ‘right middle pole’, ‘left upper pole’, or ‘right upper pole’. If multiple cancer sites are present, list all of them and separate them with a comma. If the cancer is throughout the kidney, or if it replaces the kidney, answer all three poles of the laterality being examined. ‘Superior pole’ is synonymous to ‘upper pole’, ‘inferior pole’ is synonymous to ‘lower pole’. Format your answer as one or more of these six options only. Do not provide explanations.
Prostate	
PIRADS	Tell me the maximum PI-RADS Score of all the lesions in this report. For example, if there are three lesions with PI-RADS scores of 5, 4, and 4, then you should return “5”. If there is only one lesion, return the PI-RADS score of that lesion. If there are both v1 and v2 PI-RADS scores reported, return the v2 scores. Return your answer as a JSON with the field name “overall_pirads_score”. If you cannot find the data, return “NA” in the field. Format the field as type character. Do not include units with values, and make all letters lowercase.
Laterality specific cancer (left)	Is there a lesion or cancer present in the left side of the prostate? Return your answer as “1” for “yes” or “0” for “no” in JSON format with the field name “lesion_left_side”. If you cannot find the data, return “NA” in the field. Format the field as type character. Do not include units with values, and make all letters lowercase.
Laterality specific cancer (right)	Is there a lesion or cancer present in the right side of the prostate? Return your answer as “1” for “yes” or “0” for “no” in JSON format with the field name “lesion_right_side”. If you cannot find the data, return “NA” in the field. Format the field as type character. Do not include units with values, and make all letters lowercase.
MDS	
Bone marrow	Is the following pathology report from a bone marrow biopsy rather than a FLT3 DNA report, cytogenics report, or FISH report? Please ONLY answer either “yes” or “no”. Do not provide explanations.
Untreated myelodysplastic syndrome	Is the biopsy usable? Please ONLY answer either “yes” or “no”. A sample is usable if the pathology report notes MDS is present, if the biopsy is not from an outside center or by referral, if the patient has not been pretreated, if the patient does not have AML, MPN, myelofibrosis, essential thrombocythemia, thrombocytosis, polycythemia vera, chronic myelomonocytic leukemia (CMML), or any other clonal neoplasm. Do not provide explanations or output anything else.

DCIS = ductal carcinoma in situ, IDC = invasive ductal carcinoma, ILC = invasive lobular carcinoma, FEA = flat epithelial hyperplasia, ALH = atypical lobular hyperplasia, ADH = atypical ductal hyperplasia, PI-RADS = Prostate Imaging-Reporting and Data System, FLT3 = FMS-like tyrosine kinase 3, FISH = fluorescence in situ hybridization, AML = acute myeloid leukemia, MPN = myeloproliferative neoplasms.

**Table 3:** Dataset-Level exact match accuracies for the second human Annotator, GPT-4 and Open-source LLMs.

	Second human annotator	Closed-source zero shot	Open-source zero-shot							Question-specific training	Dataset-specific training	Mixed-dataset training
		GPT-4	Llama-3-8B-UltraMedical	PMC-LLaMA 13B	Meta-Llama-3.1-8B-Instruct	Mistral-7B-Instruct-v0.3	Gemma-2-9b-it	Deepseek-r1-distill-llama-8b	Deepseek-r1-distill-qwen-7b	Meta-Llama-3.1-8B-Instruct	Meta-Llama-3.1-8B-Instruct	Meta-Llama-3.1-8B-Instruct
Breast	75.5 [70.1, 80.3]	84.0 [79.3, 87.8]	27.6 [22.8, 33.0]	4.8 [2.7, 7.5]	42.9 [37.1, 48.6]	29.3 [24.3, 34.7]	65.6 [60.2, 71.1]	72.4 [67.0, 77.6]	37.1 [31.6, 42.5]	89.1 [85.0, 92.5]	**91.5 [88.1**,** 94.6]**	90.8 [87.1, 93.5]
Kidney	83.3 [73.6, 90.3]	80.6 [70.8, 88.9]	44.4 [33.3, 56.9]	23.6 [15.3, 34.7]	75.0 [63.9, 84.7]	72.2 [61.1, 81.9]	12.5 [6.9, 22.2]	66.7 [55.6, 76.4]	43.1 [31.9, 54.2]	80.6 [70.8, 88.9]	**87.5 [77.8**,** 94.4]**	73.6 [62.5, 83.3]
Prostate	83.1 [76.2, 88.5]	**93.1 [87.7**,** 96.9]**	75.4 [67.0, 82.3]	21.5 [15.4, 29.2]	28.5 [20.8, 36.9]	76.9 [68.5, 83.1]	84.6 [77.7, 90.0]	80.8 [73.1, 86.9]	54.6 [45.4, 63.1]	90.8 [85.4, 94.6]	91.5 [86.2, 95.4]	90.8 [84.6, 94.6]
MDS	85.8 [80.7, 89.9]	86.7 [81.7, 90.8]	9.2 [6.0, 13.8]	17.0 [12.4, 22.5]	76.6 [70.6, 82.1]	16.1 [11.5, 21.2]	45.9 [39.4, 52.3]	7.3 [4.1, 11.5]	26.6 [21.1, 32.6]	86.7 [81.7, 90.8]	**89.4 [84.9**,** 93.1]**	86.7 [81.7, 90.4]
Average	81.9 ± 3.9	86.1 ± 4.6	39.1 ± 24.4	16.7 ± 7.3	55.8 ± 20.7	48.6 ± 26.4	52.1 ± 26.7	56.8 ± 29.0	40.4 ± 10.1	86.8 ± 3.9	**90.0 ± 1.7**	85.5 ± 7.1

Dataset-level exact match accuracies are followed by 95% confidence intervals in brackets. This metric evaluates the ability of each model to correctly extract all components from a report. We report the average exact match accuracy across all four datasets, in addition to standard deviation, in the “Average” row.

Significant vlaues are in bold.

Our manual review revealed that on average across all datasets, 48.7% of disagreements stemmed from human error and 39.4% from inherent ambiguity in the pathology reports. In the breast dataset, many human errors were due to omission of the lymph node in the specimen source, which are often buried within long reports describing multiple breast samples. In the kidney dataset, left-right labeling errors were common. In the MDS dataset, many errors seemed to stem from the complexity of the reports. Annotators have to extract multiple pieces of information from long, detailed narratives. More detailed metrics are shown in Supplementary Fig. S2.

### LLM performance

**Fig. 4:**
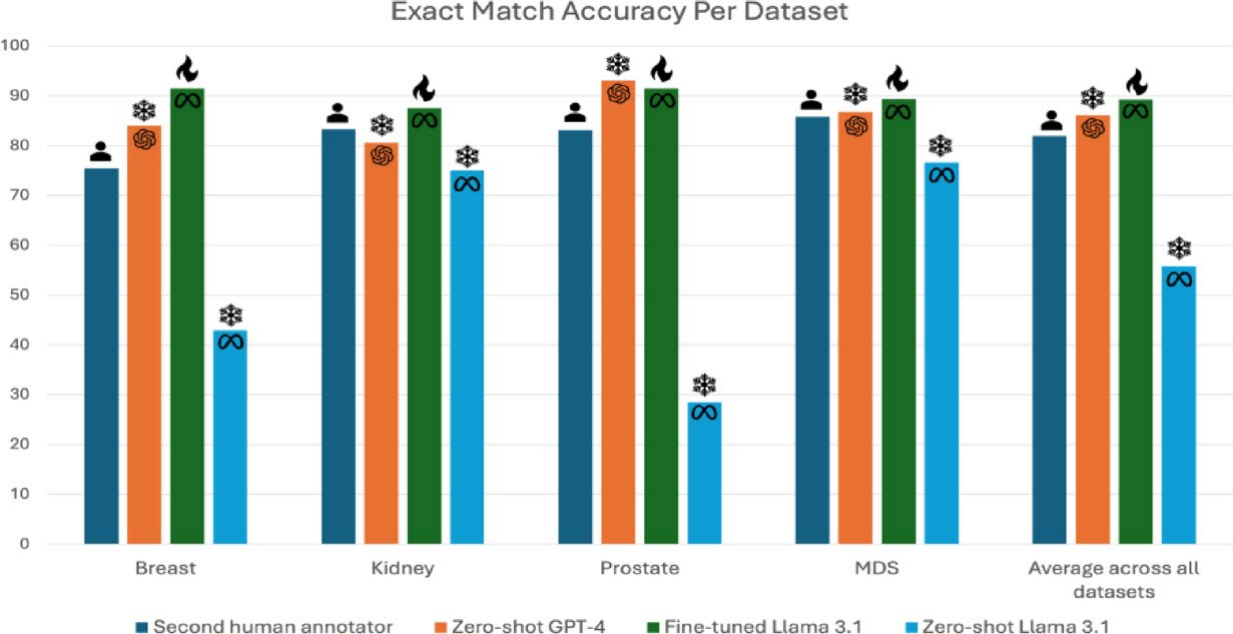
Summarized dataset-level results. Bar graph shows exact match accuracy for each dataset. Selected models include the second human annotator, zero-shot GPT-4, and fine-tuned and zero-shot Llama 3.1 8B. The human, snowflake, and flame icons represent the human, zero-shot, and fine-tuned models respectively.

We compare model accuracies to the accuracies of independent second human annotators using non-inferiority tests. We consider a model to have obtained “human-level performance” if it meets the accuracy of our second human annotator. When leveraging dataset-specific fine-tuning, Llama-3.1 8B obtained exact-match accuracies of 91.5 (95% CI 88.1, 94.6), 87.5 (95% CI 77.8, 94.4), 91.5 (95% CI 86.2, 95.4), 89.4 (95% CI 84.9, 93.1) in the breast, kidney, prostate, and MDS test set, respectively. This model obtained higher accuracies than the second human annotator in all four datasets, and the result was statistically non-inferior in all test sets (*p* < 0.001). These results are summarized in Fig. 4, and we report additional detailed metrics by data field in Table 4. We report additional metrics, including average accuracies and precision, recall, and F1 scores, in Supplementary Tables S3, S4, and S5.

**Table 4:** Detailed comparison of fine-tuned Llama 3.1 to second human Annotator.

	Fine-tuned Llama 3.1 8B	Second human annotator	Non-inferiority *p*-values
Breast			
Anatomic Site	99.0 [97.3, 99.7]	87.8 [84.0, 91.2]	< 0.001
Diagnosis	95.6 [92.9, 97.6]	92.9 [89.5, 95.2]	
Laterality-specific subtyping (left)	99.3 [97.6, 100.0]	94.2 [91.2, 96.3]	
Laterality-specific subtyping (right)	96.6 [93.9, 98.3]	95.2 [92.5, 97.3]	
Overall exact match	91.5 [87.8, 94.2]	75.5 [70.1, 80.3]	
Kidney			
Cancer Diagnosis	98.6 [93.1, 100.0]	93.1 [86.1, 97.2]	< 0.001
Cancer Anatomic Site	87.5 [77.8, 94.4]	86.1 [76.4, 93.1]	
Overall exact match	87.5 [77.8, 94.4]	83.3 [73.6, 90.3]	
Prostate			
PIRADS	97.7 [93.8, 99.2]	91.5 [85.4, 95.4]	< 0.001
Cancer (left)	96.2 [91.5, 98.5]	93.1 [87.7, 96.9]	
Cancer (right)	96.2 [91.5, 98.5]	95.4 [90.8, 98.5]	
Overall exact match	91.5 [85.4, 95.4]	83.1 [76.2, 88.5]	
MDS			
Bone marrow	91.7 [87.6, 95.0]	92.7 [88.5, 95.9]	< 0.001
Untreated Myelodysplastic Syndrome	96.9 [93.3, 99.0]	92.2 [87.6, 95.3]	
Overall exact match	89.4 [84.9, 93.1]	85.8 [80.3, 89.9]	

Data are exact match accuracies with 95% confidence intervals in brackets. Overall exact match accuracy measures if exactions across all tasks for a particular report were correct. The p-values are calculated with non-inferiority margins based on the size of each test set, 80% power, and a significance threshold of 0.05. This resulted in non-inferiority margins of 6.3%, 13.5%, 8.2%, and 5.9% for breast, kidney, prostate, and MDS, respectively.

Without fine-tuning (i.e. zero-shot), open-source LLMs obtain significantly worse performance than our second human annotator, who had an average exact match accuracy of 81.9%. DeepSeek-R1-Distill-Llama-8B obtains the highest overall performance, with an average exact match accuracy of 56.8% and 72.4 (95% CI 67.0, 77.6), 66.7 (95% CI 55.6, 76.4), 80.8 (95% CI 73.1, 86.9), and 7.3 (95% CI 4.1, 11.5) on breast, kidney, prostate and MDS, respectively. Llama-3.1 8B obtained similar zero-shot performance averaging 55.8 over the four datasets, and 42.9 (95% CI 37.1, 48.6), 75.0 (95% CI 63.9, 84.7), 28.5 (95% CI 20.8, 36.9), and 76.6 (95% CI 70.6, 82.1) for each dataset. Critically, Llama-3.1 8B obtained this performance without the use of any intermediate reasoning steps and, hence, was readily amenable to further fine-tuning. Gemma-2-9B and Mistral-v0.3 7B both obtained worse performance than Llama-3.1 8B, with average exact match accuracies of 52.1% and 48.6%, respectively. We found that LLama-3-8B-UltraMedical and PMC-LLaMA 13B both performed worse than Llama 3.1 8B, with 39.1 and 16.7 average exact match across datasets.

Llama-3.1 8B obtained qualitatively similar results across fine-tuning settings, obtaining average exact match accuracies ranging from 85.5% to 90.0% compared to 81.9% by second human annotators. These results suggest that researchers can obtain human-level results if they annotate data for a single task (i.e., question-specific finetuning); alternatively, institutions can train a single model to support all tasks (i.e., mixed-dataset finetuning) to obtain human-level results. Dataset-specific fine-tuned Llama-3.1 8B significantly outperformed its zero-shot counterparts across all datasets (*p* < 0.05), with high average precision and recall of 93.9 and 91.0. We visualize model predictions for clinical tasks in Supplementary Figure S2. GPT-4, which received no fine-tuning, obtained an average exact match accuracy of 86.1% across the four datasets. GPT-4 was non-inferior (*p* < 0.001) to the second human reader in breast, prostate, and MDS test sets. GPT-4 did not obtain non-inferior accuracy relative to the second human annotator (80.6% vs. 83.3%, *p* = 0.09) in the kidney test set.

### Learning curve analysis and hyperparameter sensitivity

**Fig. 5:**
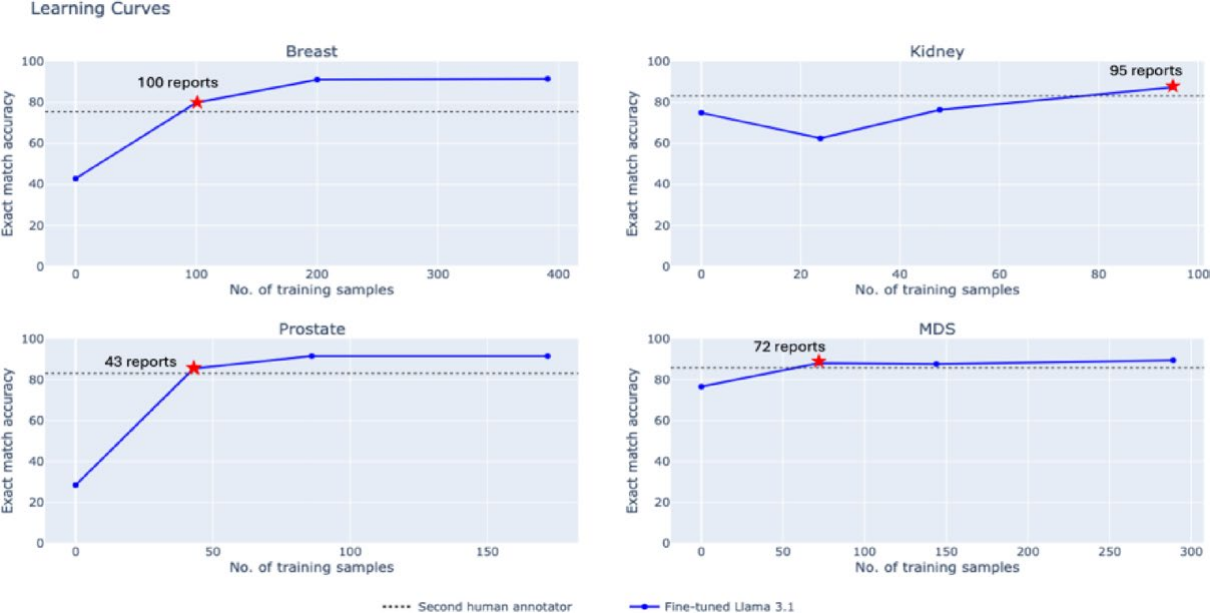
Learning curves analysis across all four datasets. Blue line graphs show the exact match accuracy for dataset-specific finetuned Llama 3.1 when leveraging 0%, 25%, 50%, and 100% of the training data. The dotted line displays the performance of the second human annotator. The stars show the number of reports used to match performance of the second human annotator.

**Fig. 6:**
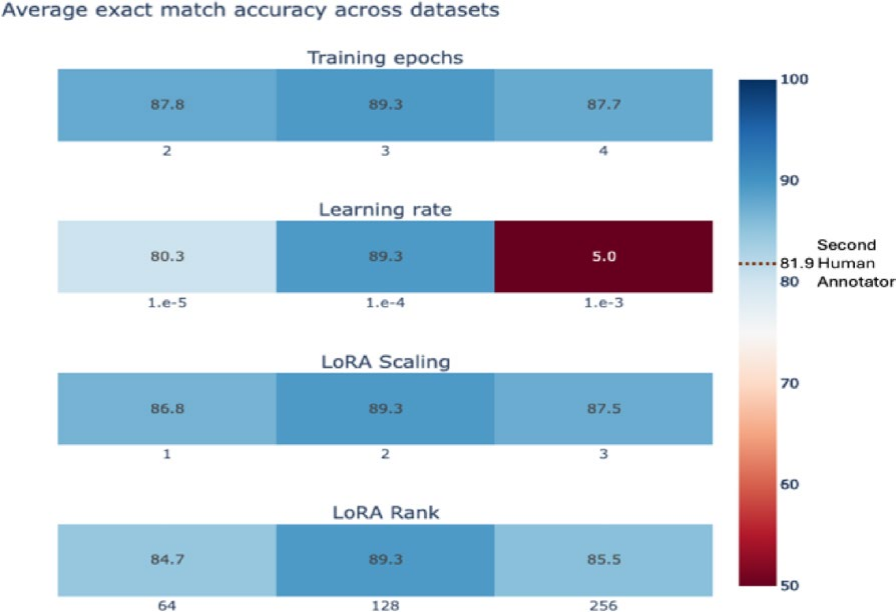
Hyperparameter sensitivity analysis for mixed-dataset fine-tuned Llama 3.1 8B. Heat maps display the average dataset-level exact match accuracy across datasets, across a range of values for each hyper-parameter. Llama 3.1 is robust to the choice of training epochs, LoRA (low-rank adaptation) scaling, and LoRA rank, but the choice of learning rate can significantly alter performance.

To understand the minimum number of training samples required to achieve human-level performance, we retrained our dataset-specific fine-tuned Llama-3.1 8B model with subsets of our training set (Fig. 5). Across each test set, we report the performance of our second human annotator on our dotted line. Our fine-tuned Llama-3.1 8B matched human performance when leveraging only 100, 95, 43, and 72 training reports for the breast, kidney, prostate, and MDS test sets, respectively. Each dataset-level model took 1–3 h to train using a single A40 GPU. At the time of writing, these experiments would cost between $0.80 to $2.40 on a private cloud. Next, we assessed the sensitivity of our models to the optimal choice of training hyper-parameters, including learning rate, number of training epochs, LoRa scaling (i.e., alpha), and LoRa rank (Fig. 6). We found that our fine-tuning results are sensitive to the choice of learning rate; while leveraging a learning rate of 1e-4 obtains a dataset-average exact-match accuracy of 89.3%, learning rates of 1e-3 and 1e-5 obtain degraded accuracies of 5.0% and 80.3%, respectively.

## Discussion

We developed Strata, a lightweight open-source library for LLM-based clinical information extraction. Strata supports a wide range of LLMs, prompt development, parameter-efficient fine-tuning, model evaluation, and deployment. Using Strata and minimal compute resources, namely a single commodity GPU (Nvidia A40) per experiment, we demonstrated that fine-tuned open-source LLMs achieve human-level performance in diverse, real-world clinical research applications across prostate MRI, breast pathology, kidney pathology, and myelodysplastic syndrome pathology reports. Our fine-tuned Llama-3.1 8B model was non-inferior to a second human annotator in all test sets. Moreover, Llama-3.1 8B demonstrated human performance leveraging only 100, 95, 43, and 72 training reports in our breast, kidney, prostate, and MDS test sets. Under the guidance of expert clinicians, Strata now supports multiple LLM-backed research databases at our institutions. This deployment reflects not just technical performance, but also clinician acceptance of the tool’s outputs in real research workflows. We demonstrate that locally hosted fine-tuned LLMs offer a practical solution for clinical information extraction, requiring limited computational and human effort; our “low-code” open-source library is designed to empower individual researchers to curate high-quality (i.e., human-level) databases at their instruction, supporting their downstream AI research.

Much of the prior work on using LLMs for clinical information extraction has focused on commercial models such as ChatGPT^26^. In our study, we found that GPT-4 demonstrated human-level zero-shot performance in three out of four test sets, namely breast, prostate, and MDS, and it was not non-inferior to our second annotator in the kidney dataset. Relying on commercial models to develop research databases poses several challenges. Using commercial models for protected health information requires institutional agreements to address privacy concerns, creating a significant access barrier. Moreover, rigorous research practices require reproducibility; as commercial LLMs are updated without user oversight, researchers cannot easily reproduce prior model queries or ensure consistent extraction quality. In contrast, our small open-source LLMs can be locally hosted, version-controlled, and easily customized for new applications.

While there has been rapid progress in adapting open-source LLMs to medical^20,21^ and general reasoning tasks^22^, we found that both biomedically fine-tuned LLMs and general reasoning models did not outperform the base instruction-tuned Llama-3.1 8B model in clinical information extraction. Specifically, we found that Llama-3-8B-UltraMedical and PMC-Llama 13B obtained substantially worse performance than Llama-3.1 8B. Leading reasoning LLMs, including deepseek-r1-distill-llama-8b, obtained similar performance to Llama-3.1 8B, despite the use of intermediate reasoning steps. In contrast, our fine-tuned Llama-3.1 significantly exceeded the zero-shot Llama-3.1 model, obtaining human-level performance.

We found that open-source LLMs address the limitations of traditional NLP techniques. Trivedi et al.^27^ previously evaluated the performance of conventional NLP techniques applied to breast pathology reports and found that performance was lower for cases over 1024 characters with an F-measure of 0.83. In contrast, we saw consistent performance across our datasets, where all reports across all test sets contained over 1024 characters. Yala et al.^6^ developed statistical machine-learning tools for parsing breast pathology reports; while the tool achieved an exact-match accuracy of 90%, it required over 6000 annotations to reach this performance. In contrast, we demonstrated that we could achieve human-level results leveraging only ≤ 100 training set reports, addressing the primary limitation of this prior work. As a flexible framework for low-code evaluation of new models, Strata complements recent advances in LLM adaptation for low-resource biomedical settings. Given Strata, valuable directions for future work include detailed investigations in targeted prompt tuning, output correction strategies, and alternative learning rate schedules.

Our study has limitations. We used datasets from two tertiary academic institutions, which may not account for variability in pathology report structures, terminology, and style across different institutions. Our clinical information extraction tasks only capture a fraction of the data elements in clinical reports. Additional work is needed to broaden our results to more tasks and institutions.

## Conclusion

Our study shows that fine-tuned open-source LLMs can achieve human-level performance while leveraging modest annotation (i.e., ≤ 100 reports) and computational resources (i.e., <$3.00). Self-hosted open-source LLMs offer a compelling and high-performance alternative to commercial LLMs. We release Strata, our lightweight library, to broaden access to these tools.

## Supplementary Information

Below is the link to the electronic supplementary material.

Supplementary Material 1

## Data Availability

The institutional data used in this study are not publicly available due to compliance with patient privacy protection but are available from the corresponding author on reasonable request.

## Code availability

The underlying code for this study is available at https://github.com/YalaLab/strata.
