# Supplementary material for: Human level information extraction from clinical reports with finetuned language models
Source: Sci Rep. 2025 Nov 24;15:45239. doi: 10.1038/s41598-025-28767-z (PMC12749435; doi:10.1038/s41598-025-28767-z)
Supplement: Supplementary file 1 — Supplementary Material 1 [file 41598_2025_28767_MOESM1_ESM.pdf]

Title: Human level information extraction from clinical reports with finetuned language models

Authors: Longchao Liu, BA [1*], Long Lian, BA [1*], Yiyan Hao, BS [2], Aidan Pace, BE[3], Elaine Kim, BS[3], Nour Homsi, BS[4], Yash Pershad, BS [5], Liheng Lai[1], Thomas Gracie, MD [6], Ashwin Kishtagari, MD [7], Peter R Carroll, MD, MPH [8], Alexander G Bick MD, PhD [5], Anobel Y Odisho, MD, MPH[8,9], Maggie Chung, MD[10^], Adam Yala, PhD[1,9,11^]

1. Electrical Engineering and Computer Sciences, UC Berkeley, 387 Soda Hall, Berkeley, CA 94720
2. Department of Bioengineering**,** University of Pennsylvania**,** 210 South 33rd St, Philadelphia, PA 19104
3. University of California San Francisco School of Medicine, 533 Parnassus Ave, San Francisco, CA 94143.
4. University of California Riverside School of Medicine, 92521 Botanic Gardens Dr, Riverside, CA 92507
5. Division of Genetic Medicine, Department of Medicine, Vanderbilt University Medical Center, 2213 Garland Avenue, Nashville, TN 37232
6. Department of Medicine, Vanderbilt University Medical Center, 2213 Garland Avenue, Nashville, TN 37232
7. Vanderbilt Ingram Cancer Center, Vanderbilt University Medical Center, 2220 Pierce Ave, Nashville, TN 37232
8. Department of Urology**,** School of Medicine, University of California San Francisco (UCSF), 1825 4th St, Box 1695, San Francisco, CA 94143
9. Computational Precision Health, UC Berkeley and UCSF, 2177 Hearst Ave, Berkeley, CA 94709
10. Department of Radiology and Biomedical Imaging**,** School of Medicine, University of California San Francisco (UCSF), 505 Parnassus Ave, San Francisco, CA 94143.
11. Department of Statistics, UC Berkeley, 367 Evans Hall, Berkeley, CA 94720

** Joint first authors*
^ Joint senior authors

Corresponding author:
Adam Yala PhD
Email: yala@berkeley.edu, Phone: 224-465-7241
Address: 2177 Hearst Ave, Berkeley, CA 94709

A1. Supplementary Methods: Detailed Dataset Construction

We designed our study to match real-world practice at academic medical centers. Across our current and prior institutions, chart reviews are conducted by research assistants trained and supervised by expert physicians. It is often infeasible to ask overworked physicians to label hundreds or thousands of clinical reports. In our case, research assistants have medical background as medical students and they were trained for each annotation task by experienced physicians with 8-11 years of post-medical school experience.

Breast Pathology Dataset

We performed a single-institution retrospective analysis of breast pathology reports from January 2019 to August 2023 using mPower Clinical Analytics. A total of 978 pathology reports were selected, including 609 reports that contained malignant diagnoses and 369 reports with benign and/or high-risk pathologies, to form a cancer-enriched dataset. High-risk pathologies included atypical intraductal epithelial proliferations (atypical ductal hyperplasia), lobular neoplasias (lobular carcinoma in situ and atypical lobular hyperplasia), flat epithelial atypia, radial scar/complex sclerosing lesion, and phyllodes tumor.

For each report, the readers annotated the sample source (right breast, left breast, right lymph node, and/or left lymph node) and diagnosis category (benign, high-risk, or cancer). If more than one pathology diagnosis was present from a sample source, the highest level (malignant > high risk > benign) was annotated. For malignant pathologies, cancer subtypes were further categorized as ductal carcinoma in situ, invasive ductal carcinoma, invasive lobular carcinoma, adenocarcinoma not otherwise specified, or other invasive malignant pathology. The dataset was split into 489/244/245 for training, validation, and testing.

Kidney Pathology Dataset

We performed a single-institution retrospective analysis of 250 randomly sampled kidney pathology reports from 2002 to 2019. For each report, the readers annotated the presence and site of renal malignancy. Malignant renal tumors included renal cell carcinoma, collecting duct carcinoma, oncocytoma, and renal medullary carcinoma. For malignant pathologies, the specific location of the cancer within the kidney was annotated as right upper pole, right middle pole, right lower pole, left upper pole, left middle pole, and/or left lower pole. If the site of malignancy was not specified, the report was annotated as “not specified.” Additionally, if the cancer was reported to involve the ureter, renal sinus, renal vein, veins, hilum, lymphatics, adrenal gland, or

capsule/perirenal fat, then “other” was annotated in addition to any site noted within the kidney. The dataset was split 95/71/72 for training, validation, and testing.

Prostate MRI Dataset

The UCSF Urology Department maintains a database of manually annotated prostate cancer MRIs from which data from 1508 reports were pulled. We identified 505 matching report texts in the EHR which were pulled for LLM analysis. Of these, 431 were from after 2015, which was when MRI reporting format became more standardized at UCSF. For each prostate MRI report, the readers annotated the presence of a lesion or cancer for both the right and left sides. The maximum PI-RADS score of all lesions in the report was recorded (e.g., "5" if the scores are 5, 4, and 4). In cases where both v1 and v2 PI-RADS scores were reported, the v2 scores were utilized. The dataset was split 172/129/130 for training, validation, and testing.

Myelodysplastic Syndrome (MDS) Dataset

We searched for the occurrence of the keywords “bone marrow” or “hematopathology” at least once in 5,053,485 patients in the Research Derivative database at Vanderbilt University Medical Center and created a cohort of 2,916 patients. From this cohort, we then only included 2,100 individuals whose chart included three or more occurrences of ICD10 codes for myelodysplastic syndrome (D46 and its components). We then excluded individuals who did not have next-generation sequencing results to create the final cohort of 724 patients. We then selected the first pathology report from these 724 patients to increase the likelihood that reports described myelodysplastic syndrome before treatment or transformation to acute myeloid leukemia. Our final dataset included 724 unique reports.

Each report was then manually reviewed by a hematologist to indicate if they were associated with a bone marrow biopsy and if they were usable. Our annotator indicated whether the specimen was from a bone marrow biopsy. Next, our annotator determined if the report described untreated MDS and was relevant for a downstream research question. Specifically, a report was considered as containing “untreated MDS” if the pathology report indicated the presence of myelodysplastic syndrome, if the biopsy was not obtained from an outside center or by referral, and if the patient had not been pretreated or diagnosed with acute myeloid leukemia, myeloproliferative neoplasm, myelofibrosis, essential thrombocythemia, thrombocytosis, polycythemia vera, chronic myelomonocytic leukemia, or any other clonal neoplasm.

Dataset Labeling

In addition to the original annotations described above, clinical reports in each test set received an additional annotation by an independent research assistant (i.e., a second human annotator) who did not participate in the original annotation process. Research assistants received training on the relevant pathology classifications and the specific criteria for annotation for each dataset.

Inter-Annotator Disagreements

From the review of annotator disagreements, we found that the majority of disagreements occurred from human error (e.g. an annotator recognized presence of cancer by correctly annotating the cancer type but inadvertently did not assign cancer presence at the breast level) or inherent ambiguity in the pathology reports (e.g. disagreement on whether tissue source for a sample from axillary tail of the breast that yielded lymphocytes should be annotated as from the breast or axillary lymph node). We report a detailed breakdown of these disagreements in Supplementary Table S4.

A2. Supplementary Methods: Response processing

We modified the template minimally for the DeepSeek models, removing the line in the template that prohibits explanations. We then extract and process the last line of the response (after the reasoning steps) using the same parsing function. Unless otherwise stated, the maximum sequence length of the model is 4096 tokens, with maximum generation length of 256 tokens.

For DeepSeek models, to accommodate the reasoning steps, we increase the maximum sequence length of the model to 8192 tokens and the maximum generation length to 1024 tokens. To benchmark all models fairly, we disregarded the DeepSeek reasoning steps between the “Think” tokens and evaluated only the final answer.

For PMC-Llama 13B, we applied the same increased allowance of maximum sequence length 8192 tokens and the maximum generation length 1024.

We include real examples of templates and model responses for the same report, given the breast source tissue question. Identifiable information is redacted. The Llama-3.1-8B model response is representative of all non-reasoning models.

Supplementary Tables

Supplementary Table S1. Modified DeepSeek prompts and corresponding answers

Model	Response
Meta-Llama-3.1-8B-Instruct	<|begin_of_text|><|start_header_id|>user<|end_header_id|><br><br>You are an experienced pathologist. Answer the question using the pathology report below. Base the answer on the report only. Do not add any additional information.<br><br>Pathology report: [REDACTED]<br><br>What are the tissue sources that are investigated in this report?<br>Your answer can include "left breast", "right breast", "left lymph node", or "right lymph node".<br>If multiple sources are present, list all of them and separate them with a comma.<br>Do not provide explanations. Do not output anything other than the four choices.<|eot_id|><|start_header_id|>assistant<|end_header_id|><br><br>My answer is: right breast.<|eot_id|>
DeepSeek-r1-distill-qwen-7b	<｜begin_of_sentence｜><｜User｜>You are an experienced pathologist. Answer the question using the pathology report below. Base the answer on the report only. Do not add any additional information.<br><br>Pathology report: [REDACTED]<br><br>What are the tissue sources that are investigated in this report?<br>Your answer can include "left breast", "right breast", "left lymph node", or "right lymph node".<br>If multiple sources are present, list all of them and separate them with a comma.<｜Assistant｜><think><br>Okay, so I need to figure out what tissue sources are investigated in this pathology report. Let me start by reading through the report carefully to understand what's being discussed.<br><br>The report mentions a patient who is a 65-year-old woman with a right breast mass. The biopsy was done on the right breast mass, which is described as a 10 o'clock mass, 4 cm from the nipple. The biopsy was ultrasound-guided and core needle biopsy. The findings were evaluated, and the diagnosis was made based on microscopic and gross examinations.<br><br>Looking at the sections, the biopsy was taken from the right breast mass, and the slides A1 and A2 were reviewed. The report also mentions that the findings were communicated to Dr. Abe by Dr. Ladwig on 11/12/20 via email. There's an addendum from [REDACTED] on 11/16/20, where he notes that the findings could represent partial sampling of a fibroadenoma or nodular adenosis, which could correlate with the imaging findings.<br><br>The clinical history section describes the mass as hypoechoic, well-circumscribed, and 5 mm in size. The gross description mentions multiple cores and fragments of soft, white tissue with abundant soft, dark red blood clots.<br><br>Now, the question is asking about the tissue sources investigated. The report doesn't mention any other tissues beyond the right breast. It doesn't talk about biopsies from the left breast, left or right lymph nodes, or any other parts of the body. The focus is solely on the right breast mass, which was the subject of the biopsy.

	So, putting it all together, the only tissue source investigated here is the right breast. There's no mention of any other areas like the left breast or lymph nodes. Therefore, the answer should be just "right breast."<br></think><br><br>The tissue sources investigated in this report are the **right breast**.<｜end_of_sentence｜>
DeepSeek-r1-distill-llama-8b	<｜begin_of_sentence｜><｜User｜>You are an experienced pathologist. Answer the question using the pathology report below. Base the answer on the report only. Do not add any additional information.<br><br>Pathology report: [REDACTED]<br><br>What are the tissue sources that are investigated in this report?<br>Your answer can include "left breast", "right breast", "left lymph node", or "right lymph node”.<br>If multiple sources are present, list all of them and separate them with a comma.<｜Assistant｜><think><br>Okay, so I need to figure out the tissue sources investigated in this pathology report. Let me start by reading through the report carefully.<br><br>The report is from the University of California San Francisco Department of Pathology. The patient is [REDACTED]. The medical record number is [REDACTED], and her date of birth is [REDACTED]. She presented for an ultrasound-guided core needle biopsy of a right breast mass.<br><br>Looking at the final diagnosis, it mentions a right breast mass, specifically at the 10 o'clock position, 4 cm from the nipple. The biopsy was performed on this mass, and the report includes details about the gross and microscopic findings.<br><br>The gross description mentions that the specimen consists of multiple cores and fragments of soft, white tissue admixed with abundant soft, dark red blood clot. The tissue is entirely submitted in cassettes A1-A4. So, the main tissue source here is the right breast mass.<br><br>I don't see any mention of lymph nodes in the report. The focus is solely on the breast tissue, specifically the right breast. There's no indication that any lymph nodes were biopsied or examined in this case.<br><br>Additionally, the addendum comments mention that the findings could represent partial sampling of a fibroadenoma or nodular adenosis, which are breast tissues. So, again, the sources are within the right breast.<br><br>Therefore, the only tissue source investigated in this report is the right breast.<br></think><br><br>The tissue sources investigated in this report are the right breast.<br><br>Answer: Right breast<｜end_of_sentence｜>

Supplementary Table S2. Rate of complete DeepSeek responses

	DeepSeek-R1-Distill-Llama-8B	DeepSeek-R1-Distill-Qwen-7B
**Breast**		
Anatomic Site	99.7	98.3
Diagnosis	98.6	95.6
Laterality specific subtyping (left)	99.7	96.6
Laterality specific subtyping (right)	99	95.2
**Kidney**		
Cancer Diagnosis	100	100
Cancer Anatomic Site	95.8	97.2
**Prostate**		
PIRADS	100	100
Laterality specific cancer (left)	100	99.2
Laterality specific cancer (right)	100	99.2
**MDS**		
Bone marrow	100	99.1
Untreated Myelodysplastic Syndrome	100	99.1
**Overall**		
**Average**	99.3	98.1

The percentages of reports that have the </think> token, indicating the generated answer is complete.

Supplementary Table S3. Detailed evaluation metrics for second human annotator

Second human annotator evaluation metrics			
Question	*Precision*	*Recall*	*F1*
**Breast**			
Anatomic Site	99.8 [99.3, 100.0]	77.6 [71.1, 83.0]	86.1 [80.6, 90.1]
Diagnosis	89.7 [83.4, 95.0]	90.5 [83.9, 95.8]	90.0 [84.6, 94.4]
Laterality specific cancer subtyping (left)	84.4 [69.7, 97.2]	91.4 [64.9, 97.5]	85.6 [65.2, 95.1]
Laterality specific cancer subtyping (right)	88.3 [66.4, 98.5]	97.2 [71.2, 98.7]	92.1 [68.8, 98.0]
Average	90.6 ± 6.6	89.2 ± 8.3	88.4 ± 3.1
**Kidney**			
Cancer Diagnosis	96.2 [91.5, 98.6]	98.5 [91.9, 100.0]	94.1 [86.2, 98.5]
Cancer Anatomic Site	85.7 [79.0, 91.9]	86.2 [77.4, 93.4]	85.6 [77.2, 93.6]
Average	91 ± 7.4	92.4 ± 8.7	89.9 ± 6
**Prostate**			
Laterality specific cancer diagnosis (left)	92.1 [84.2, 97.2]	95.9 [89.0, 98.7]	94.0 [88.9, 97.2]
Laterality specific cancer diagnosis (right)	96.1 [89.2, 98.8]	96.1 [89.5, 98.8]	96.1 [91.7, 98.6]
PI-RADS	78.7 [76.4, 79.6]	79.1 [77.1, 80.0]	78.9 [77.0, 79.8]
Average	89 ± 9.1	90.4 ± 9.8	89.7 ± 9.4
**MDS**			
Bone marrow	92.3 [88.0, 95.3]	100.0 [nan, nan]	96.0 [93.7, 97.6]
Untreated Myelodysplastic Syndrome	52.0 [32.2, 72.7]	81.2 [54.1, 95.2]	63.4 [43.8, 78.8]
Average	72.2 ± 28.5	90.6 ± 13.3	79.7 ± 23.1
**Overall**			
Average	85.4 ± 9	89.9 ± 1.3	86.7 ± 4.9

Dataset-level statistics include precision, recall, and F1 scores with 95% confidence intervals in brackets. Averages ± standard deviations per dataset are calculated across questions in each dataset, and overall averages ± standard deviations are calculated across the dataset averages.

Supplementary Table S4. Detailed evaluation metrics for Llama 3.1 with dataset-specific fine-tuning

**Dataset-specific fine-tuned Llama 3.1 evaluation metrics**			
Question	*Precision*	*Recall*	*F1*
**Breast**			
Anatomic Site	99.2 [96.7, 100.0]	99.8 [99.3, 100.0]	99.5 [98.4, 99.9]
Diagnosis	94.8 [87.4, 98.8]	87.6 [80.2, 94.0]	90.6 [84.3, 95.6]
Laterality specific cancer subtyping (left)	100.0 [nan, nan]	95.9 [66.7, 100.0]	97.8 [69.8, 100.0]
Laterality specific cancer subtyping (right)	89.5 [67.8, 99.4]	97.0 [72.1, 98.8]	92.6 [70.1, 98.4]
Average	95.9 ± 4.8	95.1 ± 5.2	95.1 ± 4.2
**Kidney**			
Cancer Diagnosis	99.3 [95.6, 100.0]	98.6 [91.4, 100.0]	100.0 [nan, nan]
Cancer Anatomic Site	87.6 [81.2, 93.0]	88.3 [81.6, 95.8]	88.0 [79.0, 94.3]
Average	93.5 ± 8.3	93.5 ± 7.3	94 ± 8.5
**Prostate**			
Laterality specific cancer diagnosis (left)	97.2 [90.8, 100.0]	95.9 [88.6, 98.8]	96.6 [92.2, 98.7]
Laterality specific cancer diagnosis (right)	96.2 [89.4, 98.8]	97.4 [91.7, 100.0]	96.8 [92.5, 98.8]
PI-RADS	98.2 [93.6, 99.6]	93.3 [85.5, 100.0]	95.1 [86.9, 100.0]
Average	97.2 ± 1	95.5 ± 2.1	96.1 ± 0.9
**MDS**			
Bone marrow	93.5 [89.2, 96.5]	97.4 [94.1, 99.0]	95.4 [92.9, 97.2]
Untreated Myelodysplastic Syndrome	91.7 [54.8, 100.0]	68.8 [41.1, 88.9]	78.6 [54.5, 92.9]
Average	92.6 ± 1.3	83.1 ± 20.2	87 ± 11.9
**Overall**			
Average	93.9 ± 2.1	91 ± 5.9	92 ± 4.1

Dataset-level statistics include precision, recall, and F1 scores with 95% confidence intervals in brackets. Averages ± standard deviations per dataset are calculated across questions in each dataset, and overall averages ± standard deviations are calculated across the dataset averages.

Supplementary Table S5. Dataset-Level Average Accuracies for the Second Human Annotator, GPT-4 and Open-source LLMs.

		closed-source zero shot	open-source zero-shot							question-specific training	dataset-specific training	mixed-dataset training
	**second human annotator**	**GPT-4**	**Llama-3-8B-UltraMedical**	**PMC-LLaMA-13B**	**Meta-Llama-3.1-8B-Instruct**	**Mistral-7B-Instruct-v0.3**	**gemma-2-9b-it**	**deepseek-r1-distill-llama-8b**	**deepseek-r1-distill-qwen-7b**	**Meta-Llama-3.1-8B-Instruct**	**Meta-Llama-3.1-8B-Instruct**	**Meta-Llama-3.1-8B-Instruct**
Breast	97.3 [96.7, 97.8]	98.4 [98.1, 98.9]	90.0 [88.8, 90.9]	75.9 [74.5, 77.2]	93.9 [93.3, 94.7]	92.1 [91.4, 93.1]	95.4 [94.7, 96.1]	96.5 [95.8, 97.1]	90.2 [88.9, 91.0]	98.8 [98.4, 99.1]	**99.0 [98.5, 99.3]**	**99.0 [98.5, 99.3]**
Kidney	94.0 [90.5, 96.8]	94.7 [91.3, 97.0]	88.5 [85.0, 91.7]	78.8 [73.0, 83.8]	93.8 [90.6, 96.3]	94.4 [91.7, 96.4]	87.6 [83.7, 90.7]	93.8 [90.9, 96.1]	85.6 [81.4, 89.1]	95.8 [93.2, 97.6]	**97.1 [95.1, 98.5]**	94.0 [90.7, 96.4]
Prostate	93.3 [90.8, 95.9]	**97.7 [95.6, 99.0]**	90.5 [86.9, 93.1]	62.8 [58.2, 66.4]	64.4 [60.3, 69.7]	91.3 [88.2, 93.8]	94.4 [91.8, 96.2]	92.8 [89.7, 95.1]	81.5 [77.2, 84.9]	96.4 [94.6, 98.2]	96.7 [94.9, 98.5]	96.2 [93.8, 97.7]
MDS	92.4 [89.7, 94.9]	93.1 [90.4, 95.4]	17.6 [14.1, 21.0]	52.1 [48.1, 56.6]	87.5 [84.2, 90.6]	53.1 [50.1, 56.2]	69.9 [66.1, 73.8]	48.4 [45.6, 50.1]	54.8 [50.8, 58.8]	92.8 [90.0, 95.1]	**94.3 [92.0, 96.3]**	93.0 [90.3, 95.3]
Average	94.2 ± 1.8	96.0 ± 2.2	71.7 ± 31.2	67.4 ± 10.7	84.9 ± 12.1	82.7 ± 17.1	86.8 ± 10.2	82.9 ± 20.0	78.0 ± 13.8	96.0 ± 2.1	**96.8 ± 1.7**	95.5 ± 2.3

Note – Dataset-level average accuracies are followed by 95% confidence intervals in brackets. This metric evaluates the ability of each model to correctly extract components from a report, averaged over the fields per a question. We report the average accuracy across all four datasets, in addition to standard deviation, in the “Average” row.

Supplementary Figures

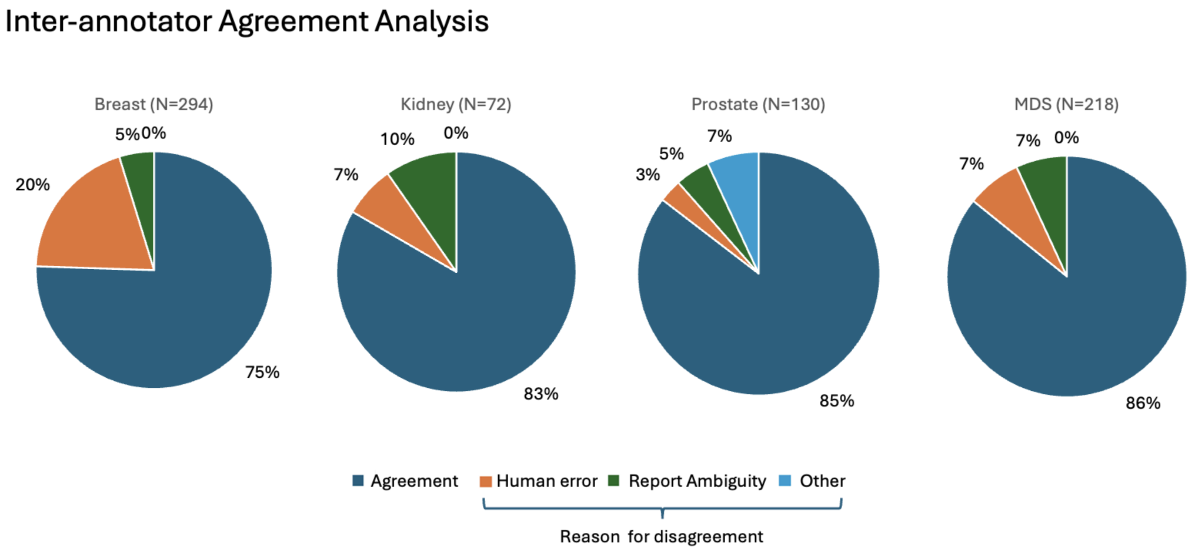

Supplementary Figure S1. Inter-annotator agreement analysis on the test set. Note – We select the "other" category for the prostate analysis when the PI-RADS score is negative, and it is unclear whether the issue stemmed from the annotators or data compilation.

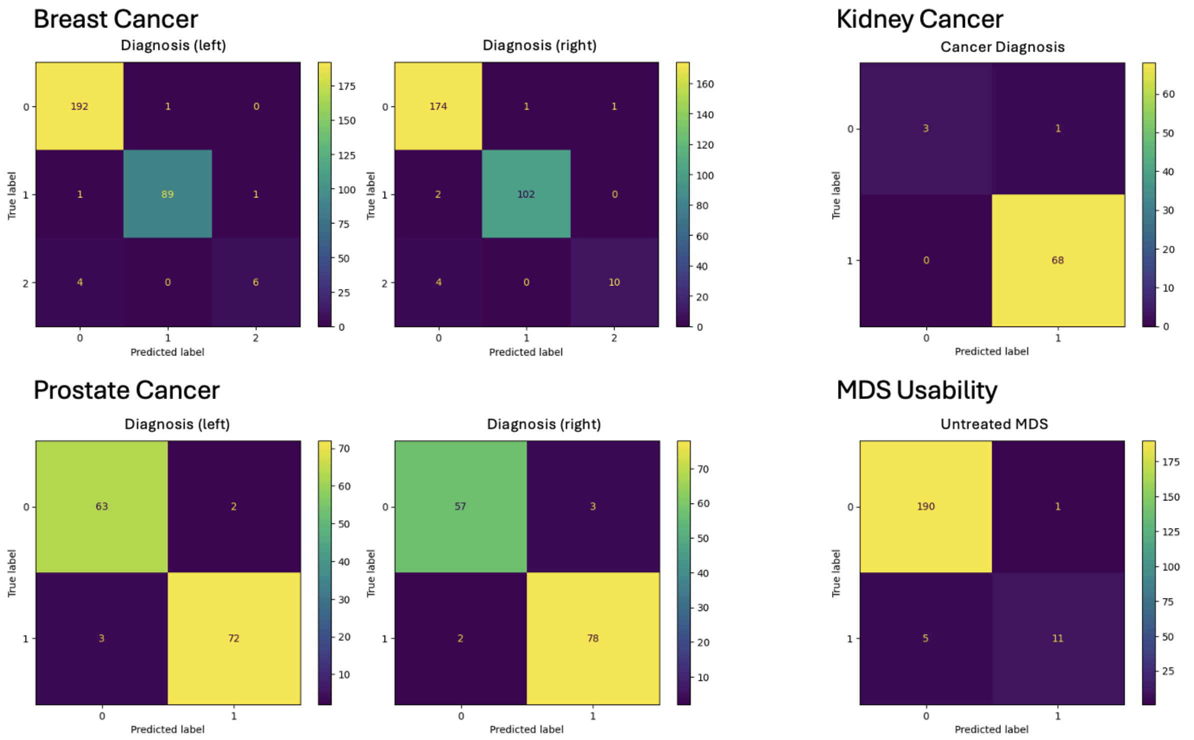

Supplementary Figure S2. Predictions from Llama 3.1 with dataset-specific fine-tuning**.** Note – for Cancer questions, response 1 indicates cancer is diagnosed, 2 indicates a benign high-risk lesion, and 0 indicates there is no cancer. For usability, a sample is usable if the pathology report notes MDS is present, if the biopsy is not from an outside center or by referral, if the patient has not been pretreated, if the patient does not have AML, MPN, myelofibrosis, essential thrombocythemia, thrombocytosis, polycythemia vera, chronic myelomonocytic leukemia (CMML), or any other clonal neoplasm.
